# Burden of Infectious Diseases, Substance Use Disorders, and Mental Illness among Ukrainian Prisoners Transitioning to the Community

**DOI:** 10.1371/journal.pone.0059643

**Published:** 2013-03-19

**Authors:** Lyuba Azbel, Jeffrey A. Wickersham, Yevgeny Grishaev, Sergey Dvoryak, Frederick L. Altice

**Affiliations:** 1 Ukrainian Institute on Public Health Policy, Kyiv, Ukraine; 2 AIDS Program, Section of Infectious Diseases, Department of Internal Medicine, Yale University School of Medicine, New Haven, Connecticut, United States of America; 3 Division of Epidemiology of Microbial Diseases, Yale University School of Public Health, New Haven, Connecticut, United States of America; Public Health Agency of Barcelona, Spain

## Abstract

**Background:**

The epidemics of incarceration, substance use disorders (SUDs), and infectious diseases are inextricably intertwined, especially in the Former Soviet Union (FSU). Few objective data documenting this relationship regionally are available. We therefore conducted a comprehensive, representative country-wide prison health survey in Ukraine, where one of the world’s most volatile HIV epidemics persists, in order to address HIV prevention and treatment needs.

**Methods:**

A nation-wide, multi-site randomly sampled biobehavioral health survey was conducted in four Ukrainian regions in 13 prisons among individuals being released within six months. After consent, participants underwent standardized health assessment surveys and serological testing for HIV, viral hepatitis, and syphilis.

**Results:**

Of the 402 participants (mean age = 31.9 years), 20.1% were female. Prevalence of HIV, HCV, HBV, and syphilis was 19.4% (95% CI = 15.5%–23.3%), 60.2% (95% CI = 55.1%–65.4%), 5.2% (95% CI = 3.3%–7.2%), and 10% (95% CI = 7.4%–13.2%), respectively, with regional differences observed; HIV prevalence in the south was 28.6%. Among the 78 HIV-infected inmates, 50.7% were unaware of their HIV status and 44 (56.4%) had CD4<350 cells/mL, of which only five (11%) antiretroviral-eligible inmates were receiving it. Nearly half of the participants (48.7%) reported pre-incarcertion drug injection, primarily of opioids, yet multiple substance use (31.6%) and alcohol use disorders (56.6%) were common and 40.3% met screening criteria for depression.

**Conclusions:**

This is the only such representative health study of prisoners in the FSU. This study has important implications for regional prevention and treatment because, unlike elsewhere, there is no recent evidence for reduction in HIV incidence and mortality in the region. The prevalence of infectious diseases and SUDs is high among this sample of prisoners transitioning to the community. It is critical to address pre- and post-release prevention and treatment needs with the development of linkage programs for the continuity of care in the community after release.

## Introduction

By 2009, HIV incidence globally had decreased by 19%, yet Eastern Europe and Central Asia remain at the center of one the world’s most rapidly expanding HIV epidemics, with a 24% increase in new HIV cases. [1] This regional HIV epidemic manifests itself primarily among people who inject drugs (PWIDs), but there is evidence for a transitioning epidemic. [2] Ukraine and Russia account for 90% of the region’s infections [3] while Ukraine has one of the most volatile HIV epidemics in the world, with 1.63% of the adult population currently living with HIV/AIDS (PLWHA) [4]–a number that is estimated to double by the year 2014. [5] HIV infection among PWIDs accounts for 10% of all HIV infections globally, but 33% outside of Subsaharan Africa, [6] suggesting an altogether different regional epidemic that needs special prevention and treatment needs.

Due to high incarceration rates among PWIDs, HIV is often concentrated within prisons, yet definitive data are lacking from well-conducted serosurveillance studies in Eastern Europe. Moreover, while incarceration itself can lead to increased risk for HIV, [7] prisons can serve as important sentinel surveillance sites for detection and treatment and can be leveraged to introduce HIV risk reduction interventions. [8], [9] Ukraine, similar to other countries grappling with a transitional epidemic, houses a large percentage of prisoners incarcerated for crimes associated with increased HIV risk including commercial sex work and substance use disorders (SUDs). In Ukraine, 14.3% of prison sentences are for offenses related to narcotics. [10] Although the U.S. has the highest incarceration rate worldwide, five former Soviet Union (FSU) states–including Ukraine with 347 incarcerated per 100,000 population–report rates that are among the top ten in the world. [11] In 2011, there were 6,069 officially registered HIV-infected prisoners in Ukraine’s penitentiary system. [12] Official prison reports vary with regard to the proportion of prisoners with drug dependence, and range from 33.8% to 64.3%. [13] Precise information on patterns of HIV in prisons is hard to obtain, especially from lower and middle-income countries, which dominate the region. [14] Ukraine, as with most FSU countries, has not harnessed the criminal justice system (CJS) to reduce HIV-related transmission, morbidity, and mortality. An effective approach in this regard could be expanded to the countries of Eastern Europe and Central Asia where HIV infection among PWIDs prevails. [15], [16].

Throughout the world, and specifically within Ukraine, incarceration, drug use, and HIV are inextricably linked. Systematic approaches to address incarceration as a means to curbing the HIV and substance use epidemics, however, are currently limited. This is particularly true because of a lack of scientifically rigorous data to describe the magnitude of the problem within prisons. Seroprevalence studies and risk assessments are central to global and local health planning strategies. [17], [18] Where incarceration is the prevailing policy toward PWIDs, prisons remain an important context for identification of diseases, initiation of treatment, and deployment of secondary prevention strategies. [14] At present, there are no recent HIV serosurveys among prisoners in the FSU, suggesting the need to reassess the situation–including prevalence of infectious disease, SUDs, and psychiatric conditions–all of which need diagnosis, treatment and continuity of care post-release. We therefore specifically sampled sentenced prisoners who were within six months of community release to conduct a surveillance assessment, describe a representaive population of prisoners transitioning to the community, and provide a pre-release assessment and referral to community services.

## Methods

### Ethics Statement

This study was approved by both the Institutional Review Boards at the Yale University School of Medicine and the Ukrainian Institute on Public Health Policy. Further safety assurances were provided by the Office for Human Research Protections (OHRP) in accordance with the 45 CFR 46.305(c) “Prisoner Research Certification” requirement. Participants provided their written informed consent to participate in the study. Potential participants who chose not to participate were not in any way disadvantaged.

### Study Setting

**Figure 1:**
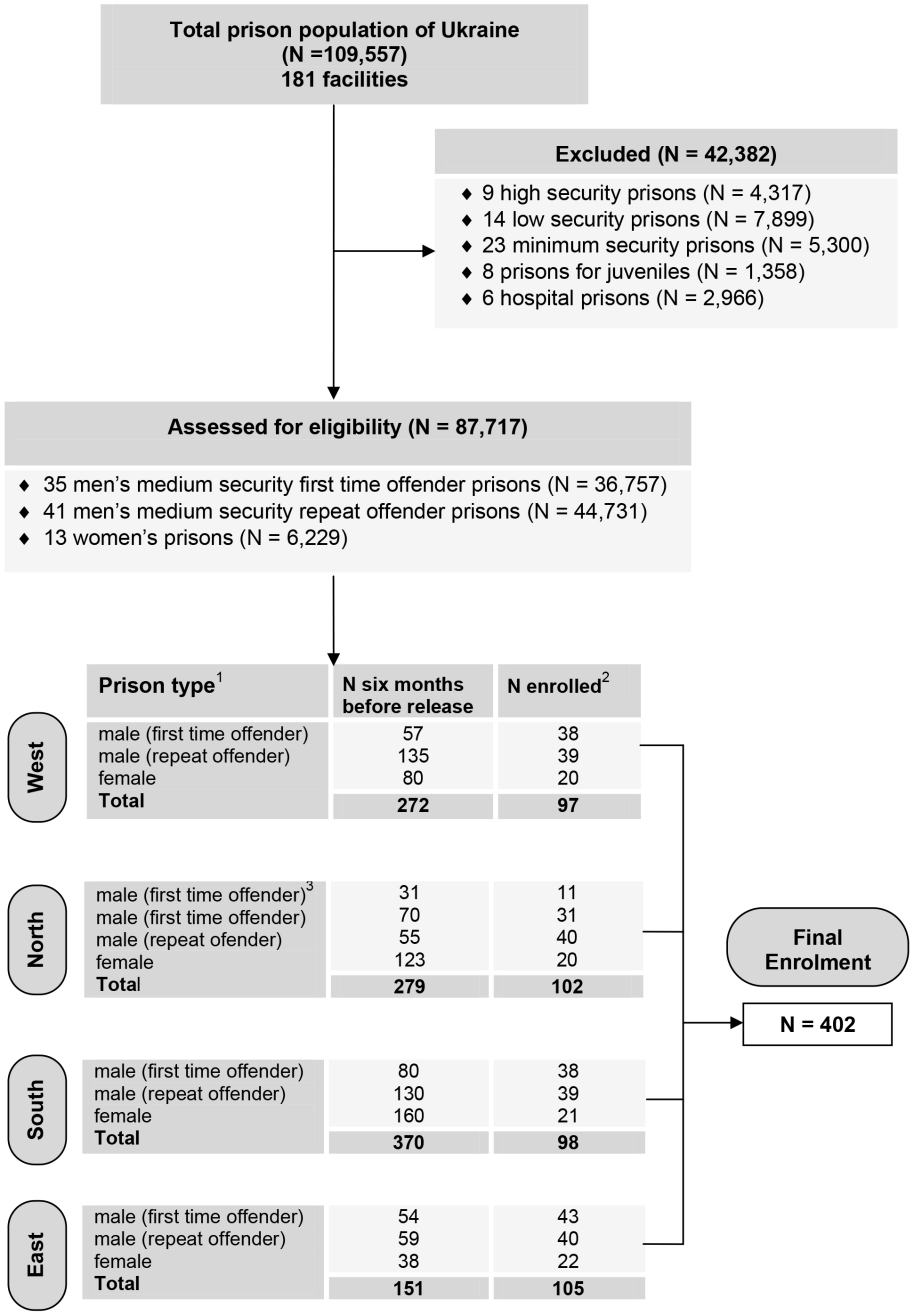
Selection of representative survey sites. Figure 1 shows the flow of participant selection. All data are current as of May 1, 2012. ^1^The administrative divisions (*oblasts*) included in each region were Kyivs’ka, Chernihivs’ka, Zhytomyrs’ka (north); Odes’ka (south); Donets’ka (east); Lvivs’ka (west). ^2^24 interviewees decided not to participate for the following reasons: already knew status (N = 7); about to transfer elsewhere (N = 6); cannot give blood (N = 5); transferred to isolation unit (N = 3); felt too sick (N = 3). ^3^Discontinued enrolment due to poor cooperation from prison staff.

A nationally representative, cross-sectional comprehensive health survey and serosurveillance assessment of infectious diseases among adult prisoners within six months of release was conducted in four Ukrainian regions between May 2011 and November 2011. The State Penitentiary Service of Ukraine oversees 181 facilities, including specialized (32 pre-trial detention, 6 medical speciality, 8 juvenile) and non-specialized (23 minimum-, 14 low-, 76 medium-, 9 high-security and 13 female-only facilities). Selection of representative survey sites are depicted in Figure 1. Only medium-security and female-only sites, which represent the majority (N = 87,717) of prisoners in Ukraine, were eligible for selection due to representational reasons and logistical restrictions imposed by the State Penitentiary Service of Ukraine. Eligible sites were categorized into one of four Ministry of Health-designated geographically representative regions of the country: east, west, north, and south. Within each region, two medium-security male prisons and one female-only prison were selected as being large, representative, and non-specialized facilities.

### Participants

A total of 402 participants met eligibility criteria and were enrolled in the study if: 1) 18 years of age or older; 2) within six months of their scheduled release date; 3) able to provide informed consent; and, 4) could read and/or speak the Russian or Ukrainian language.

Participants were selected using a random, stratified sampling strategy. [19] First, we sampled from among the 87,717 sentenced women and men in medium security prisons; two male and one female facilities in each of the four regions of Ukraine (∼100 prisoners each from the north, south, west, east region), both genders (80% men and 20% women from each region), and first-time (∼50%) and recidivist (∼50%) offenders (details in Table 1). Of the 426 inmates randomly selected for consent, 402 (94.4%) were enrolled (refer to Figure 1 description for non-participation reasons). Although women account for 13.1% of the total incarcerated population within six months of release, we modestly oversampled them to obtain more accurate representation of gender-related issues; prevalence estimates were weighted for oversampling of women.

**Table 1:** Enrolled prisoners according to region (N = 402).

Region	All female offenders	Male first time offenders	Male recidivist offenders	Number from Each Region
North	20	42	40	102
East	22	43	40	105
West	20	38	39	97
South	21	38	39	98
**Total**	**83**	**161**	**158**	**402**

### Recruitment and Enrollment

**Figure 2:**
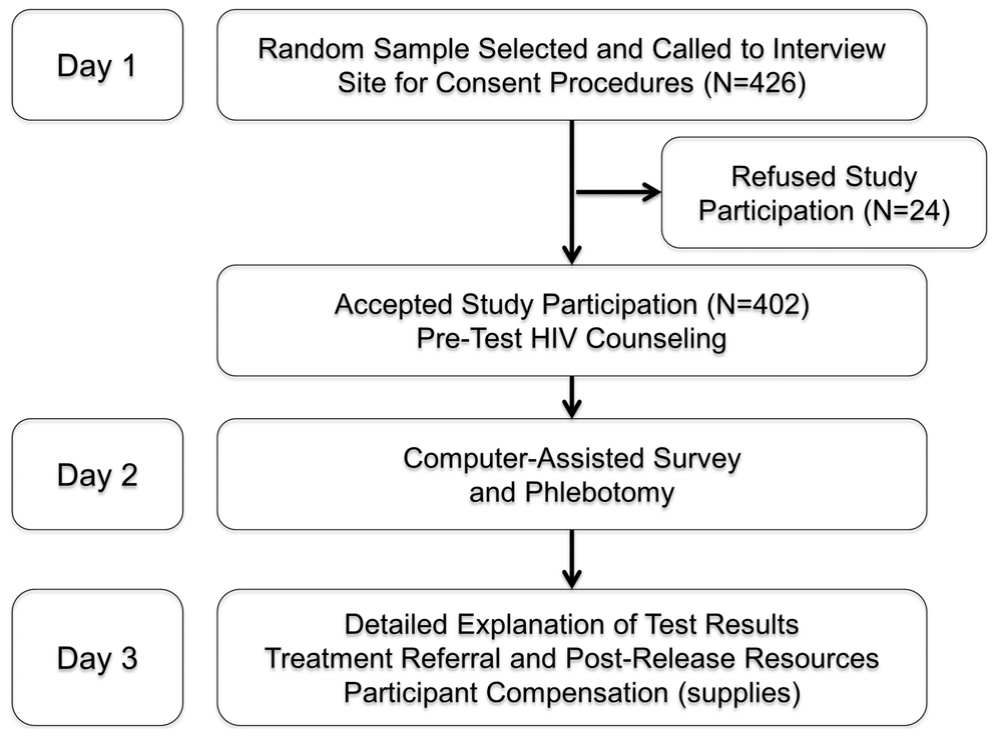
Study Activities and Procedures.

Research assistants underwent extensive training by the project coordinator. Figure 2 depicts the consecutive three-day procedures used by research staff. The State Penitentiary Service of Ukraine provided a complete list of all prisoners with an anticipated release date within six months at each facility. Upon arrival at the facility, research assistants used a random-assignment chart to select prisoners from the list provided. They met with each prisoner individually in a private room to inform them about the study and provide pre-test HIV counseling. After informed consent, participants were enrolled and assigned an anonymous identification code which was ultimately linked with their self-reported biobehavioral data and blood specimens. Participants returned the following day for the first half of the structured interviews and underwent phlebotomy. All interviews were conducted using Audio Computer-Assisted Structured Interview (ACASI) on touchpad tablet computers. ACASI was selected to reduce bias in self-reports and protect inmate confidentiality from correctional officers. Literacy is high in Ukraine (99.7%), but in the few cases that it was necessary, the research assistants read the questions verbally to those who could not read or understand them. On the third day, the participants completed the interviews, were informed of their phlebotomy results, underwent post-test HIV counseling, and were referred to medical and social services in the community according to their place of release. They were then offered hygienic and food supplies equivalent to 80 Ukrainian Hryvnia (10 USD) for their participation.

### Data Collection Content and Variables

Self-reported information included: 1) demographic characteristics; 2) criminal justice history; 3) medical history and health care utilization; 4) pre-incarceration social circumstances 5) co-morbid medical and psychiatric conditions; 6) substance use in the six months prior to incarceration and alcohol use in the year prior to incarceration (substances included opioids, sedatives, amphetamines, barbiturates, or cocaine); and, 7) sexual and drug risk behaviors in the six months prior to incarceration. Drug and sexual risk behavior information was limited exclusively to pre-incarceration data to improve accuracy and minimize recall bias. Data on drug-related risk behavior during incarceration would have resulted in mandatory reporting to prison officials and was therefore not collected. Additional standardized measures included: health-related quality of life (HRQoL) using the 36-item short-form from the Medical Outcomes Survey (SF-36) which yields a psychometrically based physical and mental health summary score; [20] hazardous drinking or more severe alcohol use disorders (AUDs) were dichotomously defined as ≥8 for men and ≥4 for women and dependent drinking was defined as ≥20 using the Alcohol Use Disorders Inventory Test (AUDIT), which has construct validity [21] and is highly correlated with AUDs; [22] depressive symptoms using the 10-item Clinical Epidemiological Survey of Depression (CES-D-10), which has high sensitivity for screening and high specificity for diagnosing major depression, [23] were measured continuously and dichotomously with a score ≥10, [24] and further characterized as moderate (10–14) and severe (>14); [25] and social support was measured continuously using a standardized scale. [26] All instruments were translated and back translated into both Russian and Ukrainian using previously described methods [27].

Phlebotomy was performed by the prison nurse. All clinical specimens, coded by the research assistant, were transported immediately to the regional Ukrainian AIDS Center for serological testing. Blood tests were performed using the commercially available One-Step Multi-Infectious Disease Rapid Test Card (InTec Products, Inc. Xiamen, China) which includes testing for Hepatitis B virus surface antigen (HBsAg), antibody to Hepatitis C virus, ELISA for HIV (1&2) test, and a rapid plasma reagin (RPR) test to *Treponema pallidum* for syphilis (RPR>1∶16 was considered positive). Initially reactive HIV serology was confirmed with the Determine™ HIV-1/2 Western Blot (Abbott Laboratories, Tokyo, Japan). All HIV seropositive participants underwent reflex CD4 T lymphocyte count assessment using FACS flow cytometry. Mandatory reporting of positive syphilis testing results to prison health authorities is statutorily required. All inmates testing positive for syphilis were referred for treatment with antibiotics. All other serology and CD4 tests were allowed to remain confidential, if requested, to reduce participation bias. After research staff provided interpretation and feedback regarding all confidential testing results to inmates, participants could elect to sign a release of medical information in order to have their results revealed to the prison health staff who could then provide additional treatment if indicated (e.g. prophylaxis for opportunistic infections if CD4<200 cells/mL). All participants received a compehensive resources guide and referral for post-release medical and community services.

### Data Analysis

Statistical analyses were performed using SPSS software for Windows (version 19.0). The *t*-test and χ^2^ for categorical and continuous variables were used where appropriate, with significance defined as *p*<0.05. Prevalence rates were examined by region, sex, age, and self-reported history of substance use during the pre-incarceration period.

## Results

### Seroprevalence of Infectious Diseases

**Figure 3:**
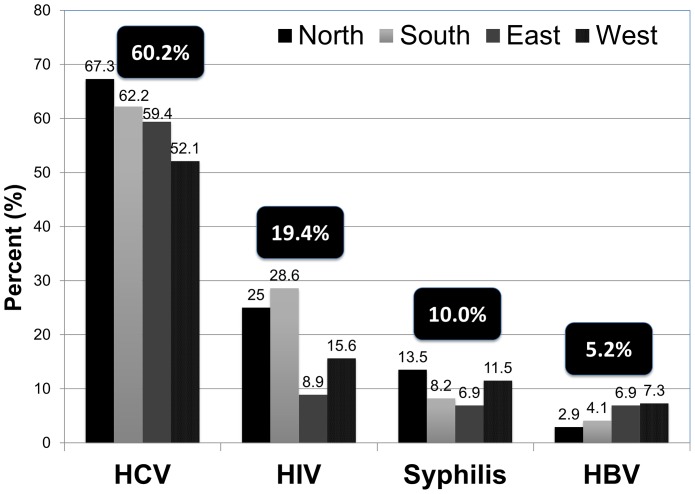
Prevalence of infectious diseases in each region (N = 402).

The prevalence of infectious diseases among Ukrainian prisoners was high (see Figure 3). Overall, 78 (19.4%: 95% confidence interval (CI) = 15.5%–23.3%) participants were HIV-infected, 242 (60.2%:95% CI = 55.1%–65.4%) were antibody positive for HCV, 21 (5.2%: 95% CI = 3.3%–7.2%) were positive for active HBV infection, and 40 (10.0%: 95% CI = 7.4%–13.2%) tested positive for syphilis. Compared to men, HIV prevalence was significantly higher among women (28.4% versus 17.3%; *p* = 0.022) and highest in the north and south regions (25.0% and 28.6%, respectively). After weighting for oversampling of women, the prevalence estimates (95% CI) were 18.6% (14.8%–22.4%) for HIV, 59.6% (54.9%–64.5%) for HCV, 5.5% (3.2%–7.7%) for HBV and 9.4% (6.6%–12.3%) for syphilis.

### Sociodemographic Characteristics

Table 2 describes the characteristics of the study sample. Though the mean age of participants was 31.9 years (range 18–58 years, *S.D.* = 8.37), recidivists, comprising nearly half of the sample (48.4%), were significantly older (35.1 vs. 28.8 years; p<0.001) than first-time offenders. Women comprised 20.1% of the sample. Compared to men, female participants were significantly older (34.0 vs. 31.4 years; *p* = 0.014). Similar to the general Ukrainian prison population where parole does not exist, average duration of incarceration was long, averaging 2.6 years.

**Table 2:** Characteristics of the prisoners (N* = *402).

	N (%)^a^
Recidivists	196 (48.8)
Mean number of previous incarcerations (range)	2.2 (0–11)
Mean current incarceration duration, years (S.D.)	2.6 (1.9)
Mean time before community release, months	2.1
*In a relationship*	
Yes	105 (26.1)
No	297 (73.9)
Mean number of arrests (S.D.)	5.4 (7.0)
Completed high school	305 (75.9)
Ukrainian or Russian ethnicity	381 (95.0)
Below poverty line	242 (60.2)
*Alcohol use disorders*	
No	167 (41.5)
Yes	229 (57.0)
Mean CES-D score (S.D.)	8.9 (5.7)
Major Depression	158 (40.3)
Social support scale score (S.D.)	2.99 (1.1)
*Health-related Quality of Life (SF-36)*	
Mean Physical Composite Score (S.D.)	47.3 (5.8)
Mean Mental Composite Score (S.D.)	38.4 (8.9)
*Mean CD4 count, cells/mL* (N = 78)	355.1
CD4>350	34 (43.6)
CD4≤350	44 (56.4)
Currently prescribed antiretroviral therapy	5 (6.4)

a Unless otherwise stated as mean (S.D.).

Key: S.D = standard deviation; CES-D = 10-item Clinical Epidemiological Scale for Depression.

Moreover, among the 78 HIV-infected inmates, slightly over half (38; 50.7%) were unaware of being HIV-infected. The regions differed by the proportion that was aware of their HIV seropositive status. In the south, only six (23.1%) had been previously diagnosed, while about half in the west (8; 53.3%) and east (5; 55.6%), and 18 (72.0%) in the north were previously informed of their HIV seropositive status (χ^2^ = 12.55, *p = *0.006 for trend). HIV prevalence was significantly higher among women (28.4% vs. 17.1%; *p*<0.001) than men. Among those who were aware of being HIV-infected, there was no difference in mean age, mean CD4 count, prevalence of AUDs, and recidivism compared to those who were unaware of their status. Participants who reported drug use in the month before incarceration (opioids, amphetamines, and PWIDs) were significantly more likely to be previously diagnosed (*p*<0.001) than those who did not use drugs.

Among HIV seropositives, mean CD4 count was 355 cells/µL (range 5–1239) with over half (56%) meeting criteria (CD4<350 cells/µL) for needing antiretroviral therapy (ART) [28] and five (6.4%) having profound immunosuppression with CD4<50 cells/µL. Only five of the 44 participants whose CD4 meet ART criteria were receiving it, though adherence for these five patients was high (mean = 97.6%; *S.D.* = 4.34). There was no difference in mean CD4 count between those participants previously informed of their HIV status and those previously uninformed (*p* = 0.281).

### Prevalence of Substance use Disorders and Depression

**Figure 4:**
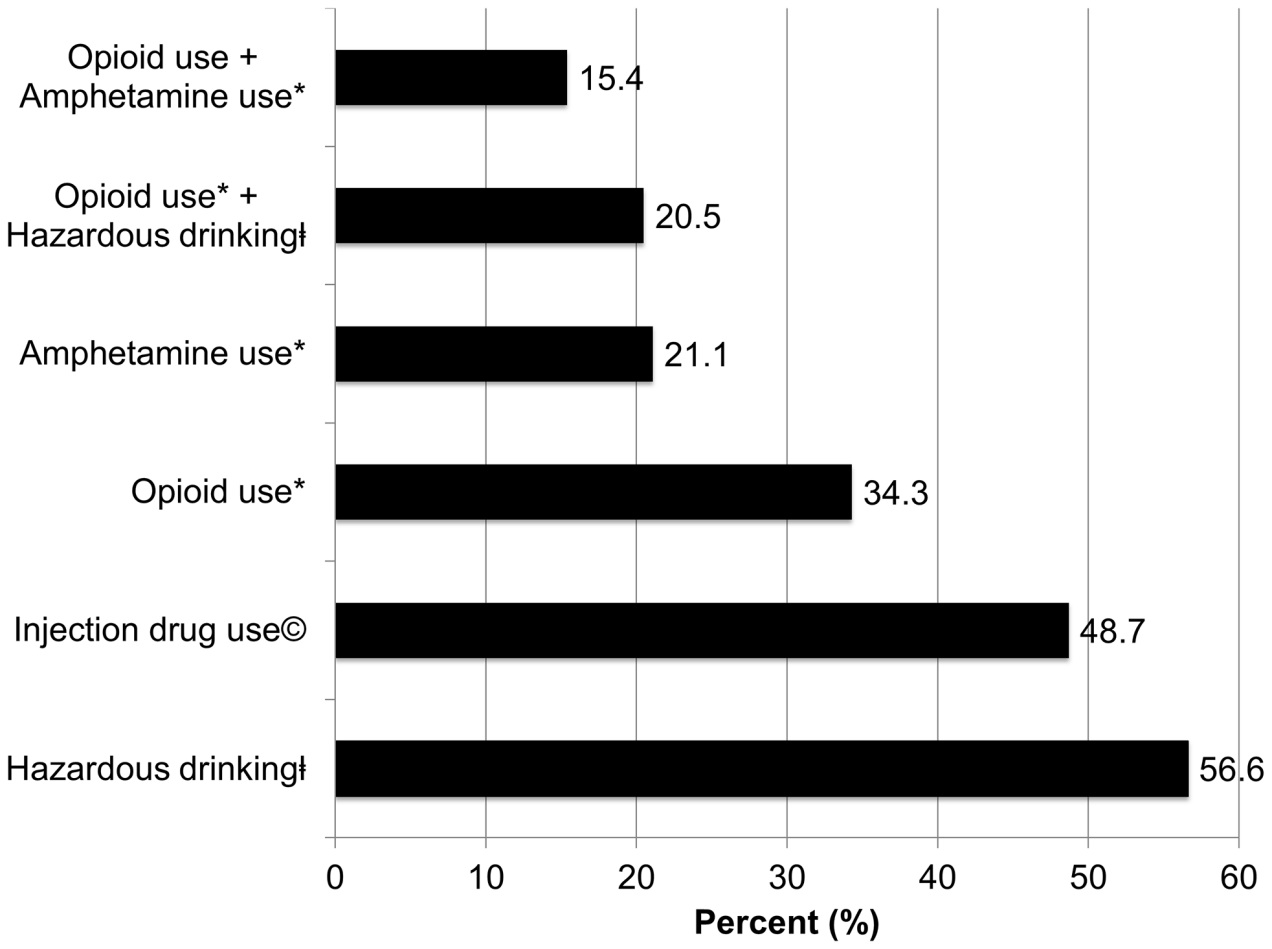
Prevalence of self-reported pre-incarceration substance use (N = 402). *Used substance at least one time in the 30 days prior to arrest. ^©^Injected drugs at least once in lifetime. ^‡^Score of ≥8 for men and ≥4 for women using the Alcohol Use Disorders Inventory Test (AUDIT).

In the 30 days prior to their arrest, alcohol and drug use was high (see Figure 4). Over half (56.6%) met screening criteria for AUDs with 18.2% of participants meeting criteria for alcohol depenence. Opioids were the most common illicit substance used in the 30 days prior to arrest (34.3%), primarily through injection, followed by amphetamines (21.1%), and sedatives (17.9%). Injection was the most common route of drug administration and nearly half (48.7%) of all participants reported injecting drugs at least once in their lifetime. Approximately a third had used more than one substance (amphetamines, opioids, sedatives, hallucinogens, cocaine, or barbiturates) in the month before they were incarcerated, most commonly opioids in combination with having an underlying AUD (20.5%) or amphetamine abuse (15.4%). The overwhelming majority (82.6%) of opioid users had used more than one substance in the month prior to their arrest and incarceration (multiple-substance use). Regional differences in drug use were observed. Opioid use was most prevalent in the north (50.0%) and amphetamine use was most common in the north and east (30.8% and 28.4%, respectively). More than half the respondents in every region except for the south had used at least one substance in the month before their most recent arrest and incarceration. 40.3% of participants met screening criteria for moderate to severe depression (see Table 2).

## Discussion

To our knowledge, this is the first published comprehensive health assessment and biobehavioral surveillance study using standardized measures from a representative sampling strategy in a prison system of a FSU country. Numerous HIV surveillance studies have been done throughout the region, but based on convenience sampling. From this study, HIV prevalence among prisoners is higher than previously reported [3] and nearly 12 times higher than in the general population. [4] In addition to HIV, this health survey also examined the prevalence of other co-morbid infectious diseases (HCV, HBV, and syphilis), SUDs and mental illness among Ukrainian prisoners. The prevalence of infectious diseases among a representative sample of prisoners who are about to transition to the community is, to our knowledge, higher than reported in any other prison settings in middle-income countries. Given the high prevalence of underlying SUDs and mental illness, both of which contribute to HIV transmission and impair HIV treatment outcomes, finding alternatives to incarceration such as expanded community-based treatment, medication-assisted therapy, drug courts, probation and parole, and structural drug policy changes would likely contribute to HIV prevention efforts since incarceration greatly disrupts lives and contributes to social instability, including increased risk behaviors. [7], [29] Until such structural interventions are deployed, however, it is crucial to implement evidence-based screening, treatment, and transitional programs that result in secondary prevention efforts and ensure continuity of care into the community [8].

The methods employed in this study maintained the anonymity of the participants while linking their epidemiologic and laboratory data, thereby reducing bias introduced by self-selected enrollment. Though a few types of prisons associated with high HIV risk were excluded from recruitment (e.g. TB and HIV infirmaries) and entry to other specialized facilities was barred by the State Penitentiary Service of Ukraine, we sampled from medium security facilities which represent 80.1% of the prison population in Ukraine. The estimates gleaned from this study, however, provide the most comprehensive assessment of disease prevalence among Ukrainian prisoners to date.

The high HIV prevalence, as well as the high prevalence of other chronic infections, attests to the need for systematic detection and treatment of diseases as this highly vulnerable population spends considerable time within and ultimately transitions through the CJS. Treatment during incarceration, particularly since the mean duration of incarceration exceeded 2.5 years, is crucial to improve individual health outcomes. Until societies decide to favor provision of evidence-based treatment in community settings over incarceration, such settings remain sentinel sites for detection, treatment, and continuity of care for medically and socially marginalized persons. [8], [30], [31] If HIV treatment as prevention efforts are to eventually prevail in reducing HIV transmission, it is critical to overcome the gaps between under-diagnosis and under-treatment of HIV among prisoners. As noted here, only half of HIV-seropositives were aware of their HIV-status and, among those, only five participants were receiving ART (6.4% of the total HIV-infected population), indicating that only one in nine HIV-infected prisoners at the end of their sentence who needed ART was receiving it. These disparities match community-derived data on ART access [32] and contrast profoundly with data from opioid substitution therapy (OST) sites which suggest that 33.1% of patients enrolled in OST programs in Ukraine are receiving ART [33].

In the second half of 2011, 18,304 prisoners were released into the community in Ukraine. [34] Using our data, we can estimate that this translates to a total of 3,550 (95% CI = 18.8%–20.0%) HIV-infected inmates or 592 newly released HIV-infected inmates each month. With half of these individuals unaware of their status, they engage in high sex and drug risk-taking behaviors which pose a significant risk of HIV transmission to the community after prison-release. [35]–[37] Furthermore, these estimates provide valuable insight into the profound underestimation of the 6,069 officially registered HIV-infected prisoners within the prison system, [12] potentially because HIV testing is not routinely available, leaving behind many who are either unaware of their status or who refuse disclosure. Multiple studies document that individuals who are unaware of their HIV status but who are informed about being HIV-infected markedly reduce HIV-related risk behaviors. [38] As such, implementing routine HIV testing should have a marked impact on HIV transmission when significantly more HIV infected people who transition through the CJS learn about their HIV status. Moreover, removing the prerequisite of registering in government name-based HIV programs for ART eligibility would increase access to life-saving treatment. [39] At a miminimum, scaling up of voluntary counseling and testing (VCT), if not changes from VCT to routine testing, and increasing ART prescription would ultimately result in increased viral suppression and, therefore, contribute signficantly to reduced HIV transmission [40].

The HIV epidemic is further exacerbated by sexually transmitted infections (STIs). Though syphilis incidence in Ukraine has not fallen significantly [41] after the socioeconomic changes in the early 1990s, [42], [43] it is clear that the high prevalence of syphilis in Ukrainian prisons (10.0%) merits increased testing and treatment, [44] perhaps with the implementation of evidence-based HIV risk reduction interventions that are documented to be effective in reducing sexual transmission.

Despite the precautions taken to preserve anonymity, the serological testing can be used to point to an even greater drug use epidemic among PWIDs than the data illustrate using exposure to HCV as an indicator of injection-related HIV risk. [45] Only about half (48.7%) of participants reported having injected drugs at least once in their lifetime. If we conservatively estimate that 67% of PWID in Ukraine are infected with HCV, [46] we would expect a 33% HCV prevalence in our sample (0.67*0.49). The prevalence of exposure to HCV in the study population, however, is much higher at 60.2%. If we take the self-reported injection history to be true, this would indicate that only 55% of the infections would be attributable to PWIDs (0.33/0.60). Since PWIDs is the primary contributor to HCV transmission n, [47] it is highly likely that drug injection is under-reported, unless there is markedly increased HCV transmission related to sex, tattooing, or blood transfusion than reported elsewhere. Moreover, among the HIV-infected participants, HCV prevalence is 92.3%, yet IDU is reported by only 71.4% of them. Of the 22 HIV-infected participants who reported never having injected drugs, 18 (81.8%) tested positive for HCV, further pointing to under-reporting of drug injection.

Similar to other reporting mechanisms, [48] our findings confirm the considerable HIV prevalence variation between regions in Ukraine. In non-correctional settings, 2011 national reports suggest that the highest HIV prevalence is recorded in the southeast regions of the country, with the eastern region exceeding the national average. [48] This study, however, shows the lowest HIV prevalence in the east (even while HCV meets the national average) and, at first glance, does not affirm national reports. Further examination of the data, however, reveals that the discordance can be explained by the average incarceration duration of 2.6 years, which provides a snapshot of the community infection rates several years ago. Data from five years ago suggested HIV prevalence among PWIDs in the east was low, but HIV incidence in the region is currently among the fastest growing in the country. [49] This attests to the volatility of the epidemic in Ukraine.

Last, this study confirms findings from other CJS that the burden of infectious diseases beyond HIV is high. [50] In this setting, however, the burden is markedly higher than reported elsewhere, particularly in representative prison-derived samples. In a study examining HIV prevalence in low- and middle-income countries, the findings from this study exceed the prevalence of HIV reported among PWIDs in 75 countries. [14] This study, therefore, has important implications for prevention on treatment and care in all countries of the FSU since this region, unlike elswhere globally, has not shown any reduction in HIV incidence and mortality in recent years. [1] Efforts in this region to not only reduce incarceration, especially for drug-related offenses, but to expand evidence-based treatment opportunities in community settings, especially OST, are urgently needed for PWIDs. [7], [9], [51] For those who do end up in prison, there remains an urgent need to screen and provide evidence-based treatment within the prison setting and continuity of care after release to the community. Such efforts are likely to significantly reduce HIV transmission in Ukraine and other FSU countries. Despite findings that evidence-based interventions can and are implemented during incarceration elsewhere, it will be important as part of the FSU HIV prevention agenda to do so within criminal justice settings and to continue them after release. Such internvetions documented to be effective include the introduction of medication-assisted therapies like methadone, buprenorphine, and extended-release naltrexone (XR-NTX) for opioid dependence [8], [52]–[54], XR-NTX for AUDs (and potentially opioid), [9], [55] and ART for treating HIV. [56] Infectious disease, mental illness, and substance use co-morbidities within the prison population can be effectively addressed through establishing treatment and linkage programs that develop a continuum of care from the highly structured prison to the chaotic community setting. Although OST was introduced in Ukraine in 2004, [57] presently, it is unavailable within criminal justice settings. XR-NTX, recently approved in Ukraine, may have particular appeal for use and benefit the large proportion of prisoners who had use multiple substances combining either alcohol or methamphetamine with opioids. The broad findings from this study provides documentation of the great burden of diseases that are prevalent in Ukrainian prisons and provides justification for introducing evidence-based interventions to assist with diagnosis, prevention, and treatment efforts as a means to reduce HIV transmission, and other infectious diseases, among PWIDs and their partners.
